# A blocking ELISA based on virus-like nanoparticles chimerized with an antigenic epitope of ASFV P54 for detecting ASFV antibodies

**DOI:** 10.1038/s41598-023-47068-x

**Published:** 2023-11-15

**Authors:** Chaohua Huang, Chenfu Cao, Zhichao Xu, Yanxing Lin, Jiang Wu, Qiaoyu Weng, Zheng Liu, Ye Jin, Peng Chen, Qunyi Hua

**Affiliations:** 1 Animal and Plant Inspection and Quarantine Center of Shenzhen Customs, Shenzhen, 518045 People’s Republic of China; 2 State Key Laboratory of Biocontrol, School of Life Science, Sun Yat-Sen University, Guangzhou, 510006 People’s Republic of China; 3 Kobilka Institute of Innovative Drug Discovery, School of Medicine, Chinese University of Hong Kong, Shenzhen, 518172 People’s Republic of China; 4 Hu Nan Project Bioscience LTD, Changsha, 410137 People’s Republic of China; 5 Shenzhen Biolove Technology CO., LTD., Shenzhen, 518110 People’s Republic of China

**Keywords:** Biotechnology, Microbiology

## Abstract

*African swine fever virus* (ASFV) is a highly lethal pathogen of domestic and wild pigs. Due to no vaccines or drugs available, early accurate diagnosis and eradication of infected animals are the most important measures for ASFV prevention and control. *Bluetongue virus* (BTV) core-like particles (CLPs) are non-infectious hollow nanoparticles assembled from the BTV VP3 and VP7 proteins, which could be used as a platform for presenting foreign epitopes. In this study, the secondary structure of BTV VP7 protein was analyzed and predicted using the IEDB Analysis resource. Based on the prediction results of the VP7 protein, the chimeric CLPs with an ASFV P54 epitope were successfully prepared through the BAC-to-BAC baculovirus expression system and sucrose gradient centrifugation. Based on the chimeric CLPs and mAb 2E4 against AFSV P54 epitope, a blocking ELISA for detecting AFSV antibodies was established, and its reaction conditions were optimized. Through comprehensive evaluation of the method, the results showed the chimeric CLPs-based blocking ELISA displayed the best detection performance, with an AUC of 0.9961, a sensitivity of 97.65%, and a specificity of 95.24% in ROC analysis. Compared with western blot and a commercial c-ELISA for detecting anti-ASFV antibodies, this method had an excellent agreement of 96.35% (kappa value = 0.911) and 97.76% (kappa value = 0.946) with the other tests, respectively. This ELISA also had high repeatability, with CV < 10%, and no cross-reaction with the serum antibodies against other swine viruses or *Orbivirus*. In brief, this was the first report on developing a blocking ELISA based on virus-like nanoparticles chimerized with an antigenic epitope of ASFV P54 for serological diagnosis of ASFV.

## Introduction

*African swine fever* (ASF), caused by *African swine fever virus* (ASFV), is a highly contagious viral disease in domestic and wild pigs of all breeds and ages^1–4^. ASF is characterized by high fever, hemorrhages in the reticuloendothelial system, and a high mortality rate approaching 100%^5,6^. On a global scale, ASF is one of the most devastating animal diseases. ASF has occurred in at least 74 countries in the five continents of Africa, Europe, Asia, America, and Oceania, severely hitting the local pig industry and causing huge economic losses^7^. In China, ASF was first reported in 2018^8^. Since then, it has spread widely and caused a great impact on the pig industry in China.

ASFV is a large, enveloped, and double-stranded DNA (dsDNA) virus with icosahedral symmetry^9^. It has a large and complex linear dsDNA genome of 170–194 bp, encoding a large number of proteins, including 68 structural proteins and more than 100 non-structural proteins^10–13^. The complex structure of ASFV and its ability to evade the immune system make it difficult to develop a safe and effective vaccine or drug for the prevention and treatment of ASF. Therefore, early accurate diagnosis and elimination of infected pigs have become an important measure of preventing and controlling ASF. At present, the methods for detecting ASF are mainly molecular biology methods and serological detection methods. Among them, the blocking or competitive ELISA method is the most common serological detection method recommended by WOAH for ASFV diagnosis. The selection of coating antigens is one of the key factors affecting the performance of blocking or competitive ELISA methods. Among structural proteins of ASFV, P54 is the expression product of the *E183L* gene, with a total length of 183 amino acids, located in the inner envelope of ASFV^14,15^. As a type II transmembrane protein, P54 plays a key role in virus rapid invasion and attachment to host cells and can induce apoptosis in the early stage of viral infection^16,17^. After ASFV infection, anti-p54 antibodies appear as early as eight days after infection, and high titers of anti-p54 antibodies would persist for several weeks^18,19^. Furthermore, the ELISA antibody detection method based on the P54 protein has high sensitivity, specificity, and stability^20,21^. Therefore, P54 is an ideal antigen for the early diagnosis and monitoring of ASFV infection.

Virus-like particles (VLPs) or virus core-like particles (CLPs) are virus-derived nanoparticles composed of one or more different proteins with the ability to self-assemble, which mimic the form and size of a virus particle but lack genetic material^22–25^. Due to their favorable characteristics such as their size and repetitive surface geometry, VLPs could be applied in serological detection as a foreign epitope presentation platform. The flexible, immunodiagnostic method based on VLP-conjugated microspheres to detect antibodies against alphavirus improved sensitivity by up to 2-logs and had faster sample-to-answer time over traditional methods^26^. This result implied that VLPs could further improve the performance of the corresponding detection methods. Bluetongue virus (BTV), an insect-vectored virus causing bluetongue disease in wild ruminants and livestock, is a member of the *Orbivirus* genus within the family Reoviridae^27^. It is a non-enveloped, icosahedral, segmented, double-stranded RNA (dsRNA) virus. As a virus-like nanoparticle (~ 65 nm), the BTV core particle (CLP) consists of 120 copies of VP3 and 780 copies of VP7^28,29^. The complete inner capsid shell of the BTV CLP comprises 120 copies of VP3, organized as 60 dimers, arranged on a T = 1 icosahedral lattice. The outer surface of the BTV CLP contains 780 copies of VP7, arranged (as 260 trimers) with T = 13 icosahedral symmetry^28–30^. The structural feature of BTV CLPs implies that CLPs have great potential for epitope display^31,32^.

Based on the above, a new type of BTV CLPs chimerized with an antigenic epitope of ASFV P54 is generated and purified in this study. Using an HRP-anti-P54 mAb as the detection antibody, a blocking ELISA based on chimeric BTV CLPs was developed. The established blocking ELISA was sensitive and specific for ASFV antibody detection, providing a new tool for ASFV surveillance.

## Materials and methods

### Cells, antibodies and serum samples

Sf9 insect cells (Invitrogen, Cat#: 11496-015) were grown in suspension at 28 °C in Sf-900 III SFM (Gibco, Cat#: 12658019) in a shaker incubator at 130 rpm rotation.

Mouse anti-ASFV-P54-mAb 2E4 and purified recombinant P54 were prepared in our laboratory^33^. The antigenic epitope recognized by mAb 2E4 was mapped to be located at a highly conserved region between amino acids (AA) 66 and 74 (DIQFINPYQ) of ASFV P54 protein. The stable hybridoma cell line of mAb 2E4 had been submitted to China Typical Culture Preservation Center for preservation (CCTCC No. C2014212). Swine antisera against porcine circovirus 2 (PCV2), classical swine fever virus (CSFV), pseudorabies virus (PRV), porcine reproductive and respiratory syndrome virus (PRRSV), swine vesicular disease virus (SVDV), and porcine epidemic diarrhea virus (PEDV) were preserved in our laboratory.

ASFV positive and negative reference sera were purchased from China Veterinary Culture Collection Centre. BTV-positive sera and EHDV-positive sera were kindly gifted by Yunnan Academy of Animal Husbandry and Veterinary Sciences. 190 pig sera were collected from different farms of the Guangdong Province of China between December 2018 and June 2019. To determine the cutoff value of the developed blocking ELISA, Werstern blot was used to detect these sera, and the results showed that there were 105 ASF-negative sera and 85 ASF-positive sera. 493 inactivated field sera samples of pigs were provided by the agricultural authorities of Guangdong Province, collected between July 2021 and December 2022.

### Construction of recombinant baculovirus transfer plasmid

For the construction of BTV CLPs chimerized with an antigenic epitope of ASFV P54, the secondary structure of BTV VP7 protein was analyzed using the IEDB Analysis resource (http://tools.immuneepitope.org/main/). On the website, protein prediction tools such as Bepipred Linear Epitope Prediction 2.0, Chou & Fasman Beta-Turn Prediction, Emini Surface Accessibility Prediction, Karplus & Schulz Flexibility Prediction, Kolaskar & Tongaonkar Antigenicity, and Parker Hydrophilicity Prediction were employed to identify the possible flexible structural areas of BTV VP7 which could be inserted with the antigenic epitope recognized by mAb 2E4. Subsequently, BTV *vp3* and chimeric *vp7* gene were synthesized by Sangon (Shanghai, China) and were constructed into a baculovirus transfer plasmid to generate a recombinant baculovirus transfer plasmid named pFastBac-Dual-vp3–rvp7 (Figure S1).

### Generation and amplification of recombinant baculoviruse

In order to co-express the BTV r*vp7* and *vp3* gene in Sf9 insect cells, a recombinant baculoviruse was constructed by the baculovirus expression system, as described with the user manual of the BAC-to-BAC baculovirus expression system. Firstly, the recombinant baculovirus transfer plasmid pFastBac-Dual-vp3–rvp7 was transformed into the competent cells DH10Bac via heat shock. Subsequently, blue-white spot screening and colony PCR were used to select bacterial colonies that had successfully transposed with the BTV *rvp7* and *vp3* genes. Secondly, the Invitrogen PureLink HiPure Plasmid DNA Purification Kit (Invitrogen, Cat#: K210016) was used to purify high-copy recombinant bacmid DNA from the bacterial culture. Thirdly, the recombinant bacmid DNA was transfected into Sf9 insect cells using Cellfectin II Reagent (Gibco, Cat#: 10362100), and the supernatant containing the recombinant baculovirus progeny was harvested until the transfected cells showed all of the signs of viral infection. Lastly, for baculovirus amplification, the P1 recombinant baculoviruses were subjected to 3 rounds of amplification in fresh Sf9 insect cells, and the viral titers were evaluated by plaque assay.

### Proteins expression in insect cells

For achieving high yields of BTV CLPs with the baculovirus expression system, a very low multiplicity of infection was used as described previously with some modifications^34^. The mid-exponential growth Sf9 cells (4 × 10^6^ cells/mL) were first diluted to 1 × 10^6^ cells/mL with fresh Sf-900 III SFM. The cells were grown to 2 × 10^6^ cells/mL and then subsequently diluted back to 1 × 10^6^ cells/mL. After growing to 2 × 10^6^ cells/mL again, the cells were inoculated with the recombinant baculovirus at a MOI of 0.001 PFU/cell and incubated at 28 °C in a shaker incubator at 130 rpm rotation.

At 72 h post-infection, the cells were analyzed by indirect immunofluorescence assay (IFA) using anti-ASFV-P54 mAb 2E4. The IFA process was developed as previously described with some modifications^35^. The cells were first fixed with pre-cold 4% paraformaldehyde for 1 h and then permeabilized with 0.1% Triton X-100 for 1 h at room temperature. After being washed with PBS, the cells were blocked with Pierce protein-free (TBS) blocking buffer (Thermo Scientific, CAT#: 37570) for 2 h at room temperature and then incubated with mAb 2E4 (1:500 dilution) overnight at 4 °C. Following three washes with PBS, the cells were incubated with rabbit Anti-Mouse IgG H&L (FITC) (Abcam, CAT: ab6724) for 30 min at 37 °C. After being washed thrice with PBS, the cells were observed with an inverted fluorescence microscope (ZEISS Axio Observer Z1).

### Chimeric CLPs purification

For large-scale production, 1 L of Sf9 cells were infected with a recombinant baculovirus expressed BTV VP3 and rVP7 at 0.001 MOI, incubated at 28 °C, and kept in suspension with shaking at 130 rpm. After 96 h p.i., the cells were harvested by centrifugation at 2700×*g* for 10 min. The cells were lysed in the precooled buffer (50 mM Tris–HCl (pH 8.0), 150 mM NaCl, 0.5% NP40 (v/v)) for 1 h. Following three freeze–thaw and ultrasonic pyrolysis cycles, the whole cell lysates were clarified by centrifugation at 8000×*g* for 10 min at 4 °C.

BTV CLPs chimerized with an antigenic epitope of ASFV P54 were purified by density gradient centrifugation using sucrose density media, as described previously with some modifications^36^. Sucrose solutions (30–60%) were prepared in a buffer containing 50 mM Tris–HCl (pH 8.0) and 150 mM NaCl and layered into gradients of 10% incremental steps. The clarified cell lysates were then layered on top of the sucrose gradients and centrifuged at 85,000×*g* at 4 °C for 3 h in a SW-41Ti rotor (Beckman Coulter). The 55–35% sucrose layers containing chimerized CLPs were harvested, diluted with the buffer solution (50 mM Tris–HCl (pH 8.0), 150 mM NaCl), centrifuged at 100,000×*g* for 1 h at 4 °C. And then, the pellets suspended with buffer solution. The purified chimeric CLPs were detected by Western blot using sheep BTV-positive sera and mAb 2E4.

### Transmission electron microscopy

The purified chimeric CLPs were adsorbed onto plastic and carbon-coated copper grids and washed twice by floating on droplets of ddH_2_O and then negatively stained with 2% (w/v) phosphotungstic acid (Solarbio, CAT#: G1870) for 2 min. The grids were examined through a transmission electron microscope (HITACHI TEM TH7700).

### Establishment of blocking ELISA based on chimeric CLPs

As described previously with some modifications^35^, a checkerboard titration was used to determine the optimal antigen-coating concentration of chimeric CLPs and the working concentration of the mAb 2E4. The purified chimeric CLPs were diluted to 0.5 μg/mL, 1.0 μg/mL, 2.0 μg/mL, 4.0 μg/mL, 8.0 μg/mL, and 10 μg/mL in 0.2 M Tris–HCl (pH 8.0) buffer and added to wells of a 96-well ELISA plate (Costar, CAT#: 42,592). The plates were then allowed to coat at 4 °C overnight, washed three times with PBST, and blocked with Pierce protein-free (TBS) blocking buffer for 1 h at 37 °C. After another three washes with PBST, 100 μL/well of twofold diluted ASFV positive and negative reference sera were separately added to the well and then incubated for 1 h at 37 °C. After three washes, 100 μL/well of serially diluted HRP-anti-ASFV-P54-mAb 2E4 in the range of 1:1 000–1:16,000 was added to the plate and incubated for 1 h at 37 °C. After three times washing, 100 μL/well of 3,3′,5,5′-Tetramethylbenzidine (TMB) substrate was added to all wells of the plate and incubated for 10 min at room temperature. By adding 2 M H_2_SO_4_, the chromogenic reaction was stopped. Subsequently, the optical density of each well at 450 nm was measured using a spectrophotometer (Biotek 800TS). The optimal chimeric CLPs-coating concentration and HRP-anti-ASFV-P54-mAb working concentration were determined according to the highest OD450 ratio of negative to positive reference serum (N/P).

Based on the optimal coating-antigen and mAb working concentrations, other important conditions of the blocking ELISA were also optimized, including suitable blocking buffers (1% BSA, 2% BSA, 5% skimmed milk, and 1% gelatin), serum incubation time (30 min, 1 h, 2 h, and 4 h), HRP-anti-ASFV-P54 mAb incubation time (30 min, 45 min, 60 min, 90 min, and 120 min) and chromogenic reaction time (5 min, 10 min, 15 min, and 20 min).

### Determination the cutoff criterion for the blocking ELISA

A total of 190 serum samples (85 inactivated ASFV positive sera and 105 ASFV negative sera) were tested by the optimized blocking ELISA based on chimeric CLPs to determine the cutoff value, diagnostic sensitivity, and diagnostic specificity of this method. The percent inhibition (PI) values of the test samples were calculated via the following formula: PI = (1 − [test sample OD/negative control OD]) × 100%. Subsequently, data were analyzed by receiver operating characteristic (ROC) analysis using GraphPad Prism software (Version 9.0), and the diagnostic sensitivity, diagnostic specificity, and Youden index of the assay were calculated. The optimal cutoff value of the blocking ELISA was then selected based on the Youden index of the assay.

### Analytic specificity and specificity evaluation of the blocking ELISA

To evaluate the analytical specificity, six polyclonal anti-sera against other swine viruses (PCV2, CSFV, PRV, PRRSV, SVAV, and PEDV) and two positive sera against other Orbivirus such as BTV and EHDV were detected by the developed blocking ELISA based on the chimeric CLPs.

Analytical sensitivity of blocking ELISA was determined by a twofold serial dilution of three positive sera representing strongly, mediumly, and weakly ASFV positive sera in the range of 1:10–1:2560.

### Repeatability and reproducibility evaluation of the blocking ELISA

To evaluate the intra-assay repeatability and inter-assay reproducibility of the developed blocking ELISA, three ASFV positive sera representing strongly, mediumly, and weakly positive sera, as well as three ASFV negative sera, were tested using the ELISA on one plate in one run or on three plates in three independent runs. Each serum was tested in quintuplicate, and the means, standard deviations (SD), and percent coefficient of variation (% CV) by calculated using SPSS software (version 26.0).

### Comparisons of blocking ELISA with western blot and commercial ELISA kit

493 inactivated field sera samples of pigs were collected from the Guangdong Province of China between July 2021 and December 2022 with unknown exposure to AFSV. These sera were detected in parallel by the developed blocking ELISA, western blot, and the commercial c-ELISA kit (IDVET: ID Screen African Swine Fever Competition ELISA). The western blot procedure was based on the method described previously with some modifications^37^. The purified recombinant ASFV-P54 was transferred to a nitrocellulose (NC) membrane by SDS-PAGE electrophoresis and a semi-dry membrane transfer instrument. The primary antibodies were pig sera samples, and the secondary antibody was rabbit anti-pig IgG H&L (HRP) (Abcam, CAT: ab6777). The degree of agreement (Kappa value) of the blocking ELISA with the western blot and commercial ELISA kit was calculated using SPSS software (version 26.0). The higher the kappa value, the better the consistency between the two methods; if the kappa value is between 0 and 0.40, the consistency between the two methods is poor; if the kappa value reaches 0.75 or above, it indicates that the consistency between the two methods is high.

### Statistical analysis

Software of GraphPad Prism (version 9) and SPSS 26.0 were used to perform ROC analysis and Youden’s J statistic. The degree of agreement (Kappa value) of the blocking ELISA with the western blot and commercial ELISA kit was calculated using SPSS software (version 26.0).

### Ethics statement

All methods are reported in accordance with ARRIVE guidelines. All methods were performed in accordance with the relevant guidelines and regulations. For animal experiments, the study protocol for all experiments was reviewed and approved by the Ethics Committee of the Animal and Plant Inspection and Quarantine Center of Shenzhen Customs.

## Results

### Construction of recombinant baculovirus transfer plasmid

**Figure 1:**
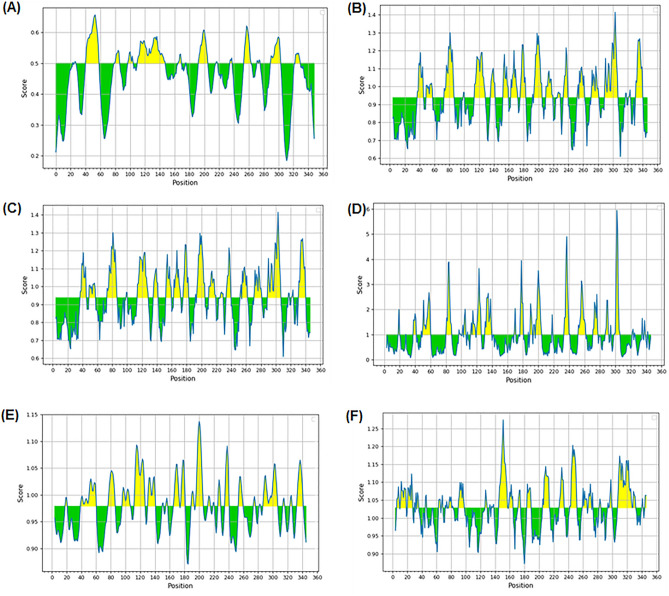
Results of possible flexible areas of BTV VP7 analyzed by IEDB. (**A**) Result of Bepipred Linear Epitop Prediction 2.0; (**B**) Result of Chou & Fasman Beta-Turn Prediction; (**C**) Result of Emini Surface Accessibility Prediction; (**D**) Result of Karplus & Schulz Flexibility Prediction; (**E**) Result of Kolaskar & Tongaonkar Antigenicity; (**F**) Result of Parker Hydrophilicity Prediction.

According to the IEDB analysis resource, the prediction results of the linear epitope, beta-turn, surface accessibility, flexibility antigenicity, and hydrophilicity of BTV VP7 (GenBank accession No. JX007928.1) were revealed (Fig. 1). After comparing and analyzing these results, it was discovered that the peptide between aa255 and aa264 could be a flexible area of the VP7 protein. And this flexible area was confirmed by other report which the antigenic epitope identified by monoclonal antibodies 20D11 and 20F10 was a linear peptide between aa259 and aa264 (QYPALT)^38^. The results of immuno-electron microscopy indicated that 20D11 and 20F10 could bind to BTV CLPs, implying that this epitope was not only present on the BTV VP7 monomer, but also on the surface of BTV CLPs. It suggests that this region might be able to display foreign antigenic epitopes.

**Figure 2:**
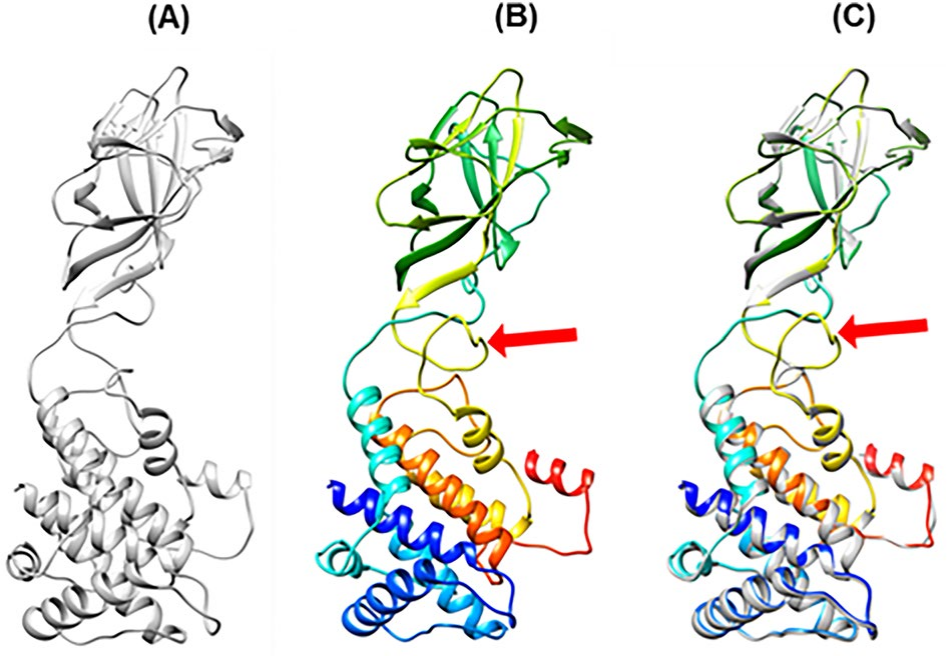
Comparison of chimeric VP7 structure with that of BTV VP7 (**A**) the VP7 structure of bluetongue virus showing in the color of grey (pdbid:1bvp); (**B**) the predicted structure of VP7 chermic with the peptide (DIQFINPYQ) recognized by anti-ASFV-P54 mAb 2E4 showing in the color of rainbow; (**C**) the two structure superimposed together. The position of the inserted peptide (DIQFINPYQ) on the structure was pointed by red arrow between aa259 and aa260.

To create chimeric VP7 protein, a short peptide (DIQFINPYQ) recognized by anti-ASFV-P54 mAb 2E4 was inserted into the flexible region between aa259 and aa264 of VP7 protein, as shown in Fig. 2. And the amino acid sequence of the rVP7 was shown in Supplement 2. In order to further understand whether the inserted amino acid sequence affects the original structure of VP7 protein, alphafold2^39^ was used to analyze and predict the structure of chimeric VP7 protein. The predicted results showed that the chimeric VP7 protein was similar to the X-ray structure of Bluetongue virus VP7^30^, possessed two domains (an antiparallel beta-sandwich upper domain, a nine-alpha-helixes formed bundle lower domains), and the insertion region of the peptide (DIQFINPYQ) still remained as a flexible area, which is conducive to the display of antigen epitope. (Fig. 2).The DNA sequences of the chimeric VP7 protein and BTV VP3 (GenBank accession No. KF664138.1) were synthesized by Sangon (Shanghai, China) and were constructed into a baculovirus transfer plasmid, generating a recombinant baculovirus transfer plasmid named pFastBacduel-vp3–rvp7.

### Expression, purification and identification of A chimeric BTV CLPs

The recombinant baculovirus, which can co-express BTV VP3 and chimeric VP7 protein with an antigenic epitope, was prepared through the BAC-to-BAC baculovirus expression system and was named rBac-vp3-rvp7. In order to achieve high yields of the chimeric CLPs, we followed the strategy of low multiplicities of infection^34^.

**Figure 3:**
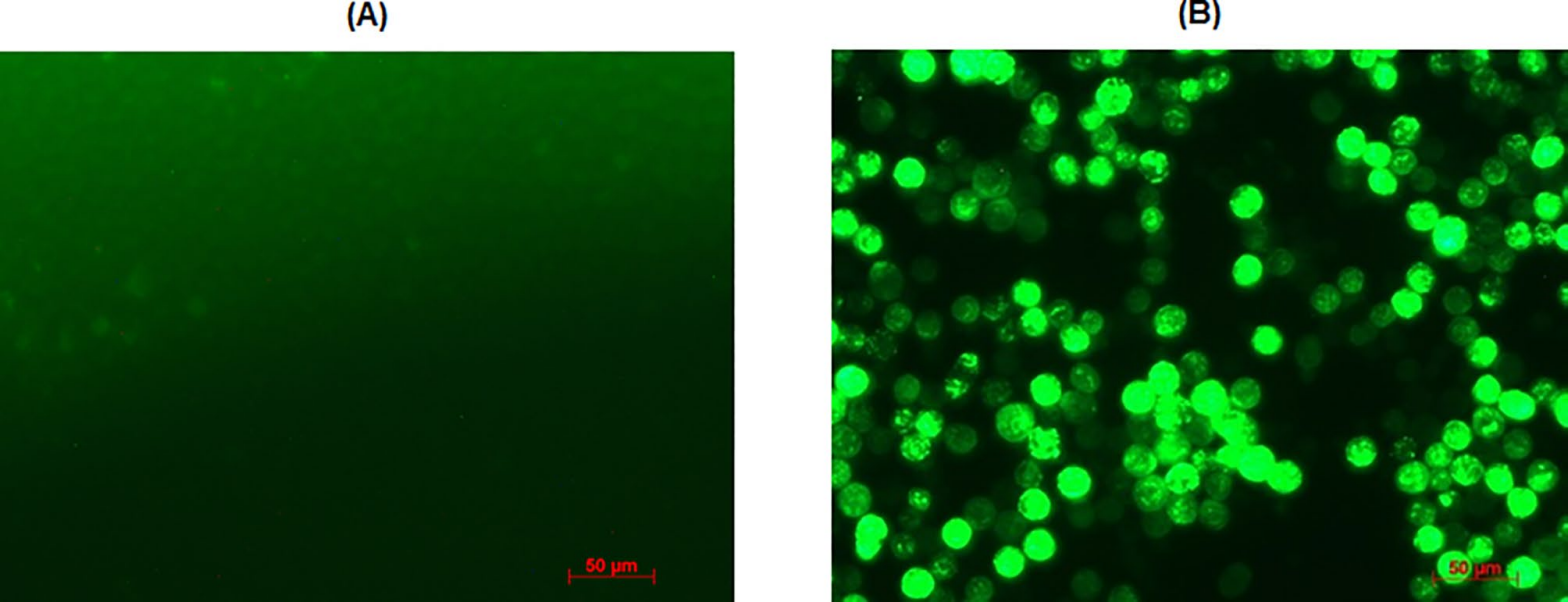
Results of IFA using mAb 2E4. (**A**) IFA result of uninfected Sf9 cells. (**B**) IFA result of infected Sf9 cells.

To investigate the expression of the chimeric VP7, the cells were analyzed by Immunofluorescence Assay (IFA) using the anti-ASFV-P54-mAb 2E4 at 72 h post-infection. As shown in Fig. 3, VP7 chimerized with the epitope recognized by the mAb 2E4 was highly expressed in the Sf9 cells.

**Figure 4:**
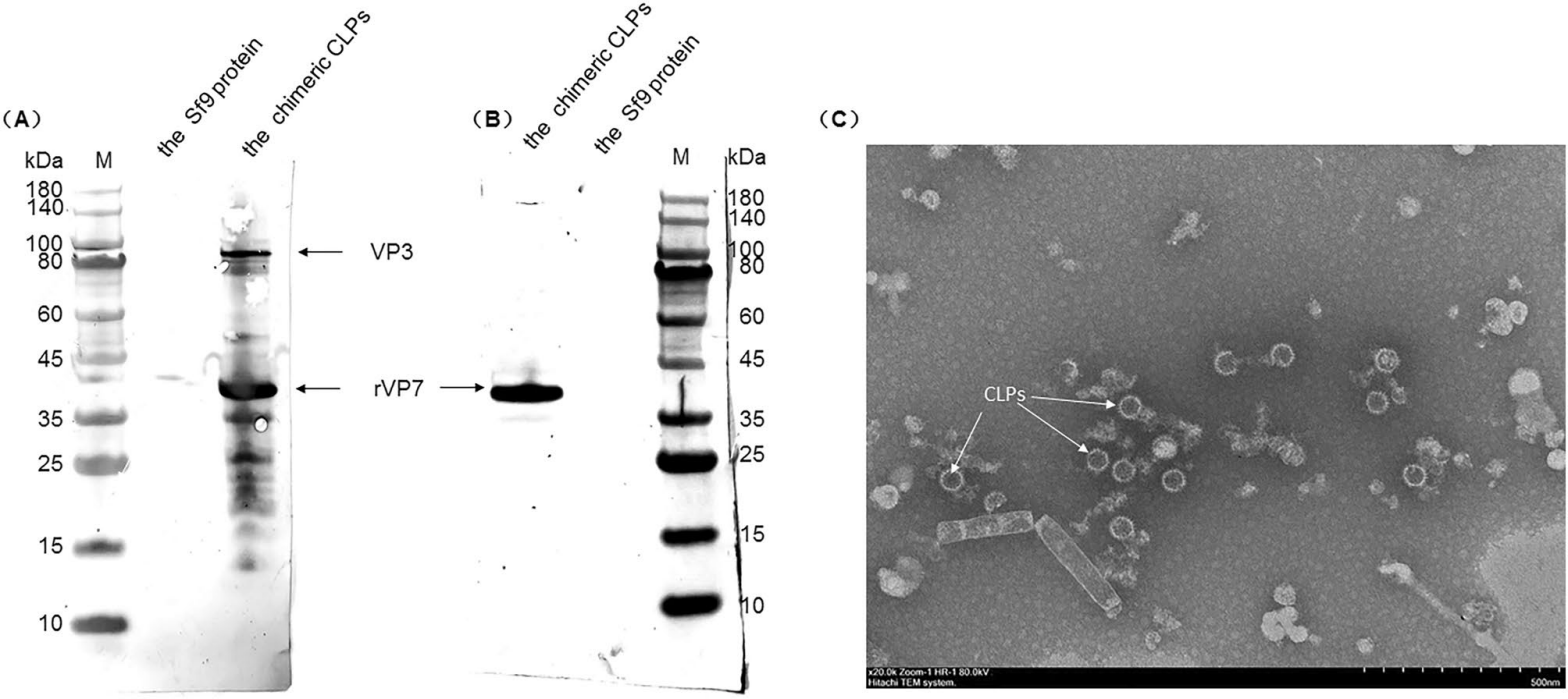
Western blot and TEM results of purified chimeric CLPs. (**A**) Western blot results of the purified chimeric CLPs using sheep BTV positive sera; (**B**) Western blot results of purified chimeric CLPs using mAb 2E4; (**C**) TEM result of chimeric CLPs Through TEM, it was observed that those chimeric CLPs have the same morphology of the native BTV CLPs, and also have a spikey coat on its outer surface which was composed of VP7 chimerized with ASFV P54 epitope.

In order to investigate whether the chimeric VP7 had assembled with BTV VP3 protein to form CLPs, a suspension culture of Sf9 cells was infected with rBac-vp3-rvp7. After 96 h, the infected cells were harvested, lysed, and purified by density gradient centrifugation using a sucrose density media. Subsequently, the product of the density gradient centrifugation was analyzed by western blot with sheep BTV-positive sera and mAb 2E4. The anti-BTV antibodies bound to protein bands corresponding in size to both the BTV VP3 (103 KDa) and rVP7 (39 KDa) proteins in the purified products (Fig. 4A). Moreover, the protein band reacted with anti-ASFV-P54 mAb 2E4 corresponded to the same position of rVP7 (Fig. 4B). This result further confirmed that the VP7 protein had been successfully chimerized with the ASFV P54 epitope recognized by mAb 2E4.

By negatively stained with 2% (w/v) phosphotungstic acid, the purified product of density gradient centrifugation was examined using a transmission electron microscope (TEM). Under the TEM, a large number of particles with a diameter of approximately 65 nm were observed in the purified product (Fig. 4C). These particles had an icosahedral appearance with a spikey coat, consistent with the native BTV CLPs reported by other researchers^40,41^. Based on the results of IFA, western blot, and TEM, the insertion of the short peptide (DIQFINPYQ) into the region of VP7 between aa259 and aa264 did not affect the expression of the VP7 protein. Meanwhile, when the chimeric VP7 protein was co-expressed with VP3 protein, it could self-assemble to form a mass of rCLPs with complete morphology.

### Establishment and optimization of the chimeric CLPs-based blocking ELISA

**Figure 5:**
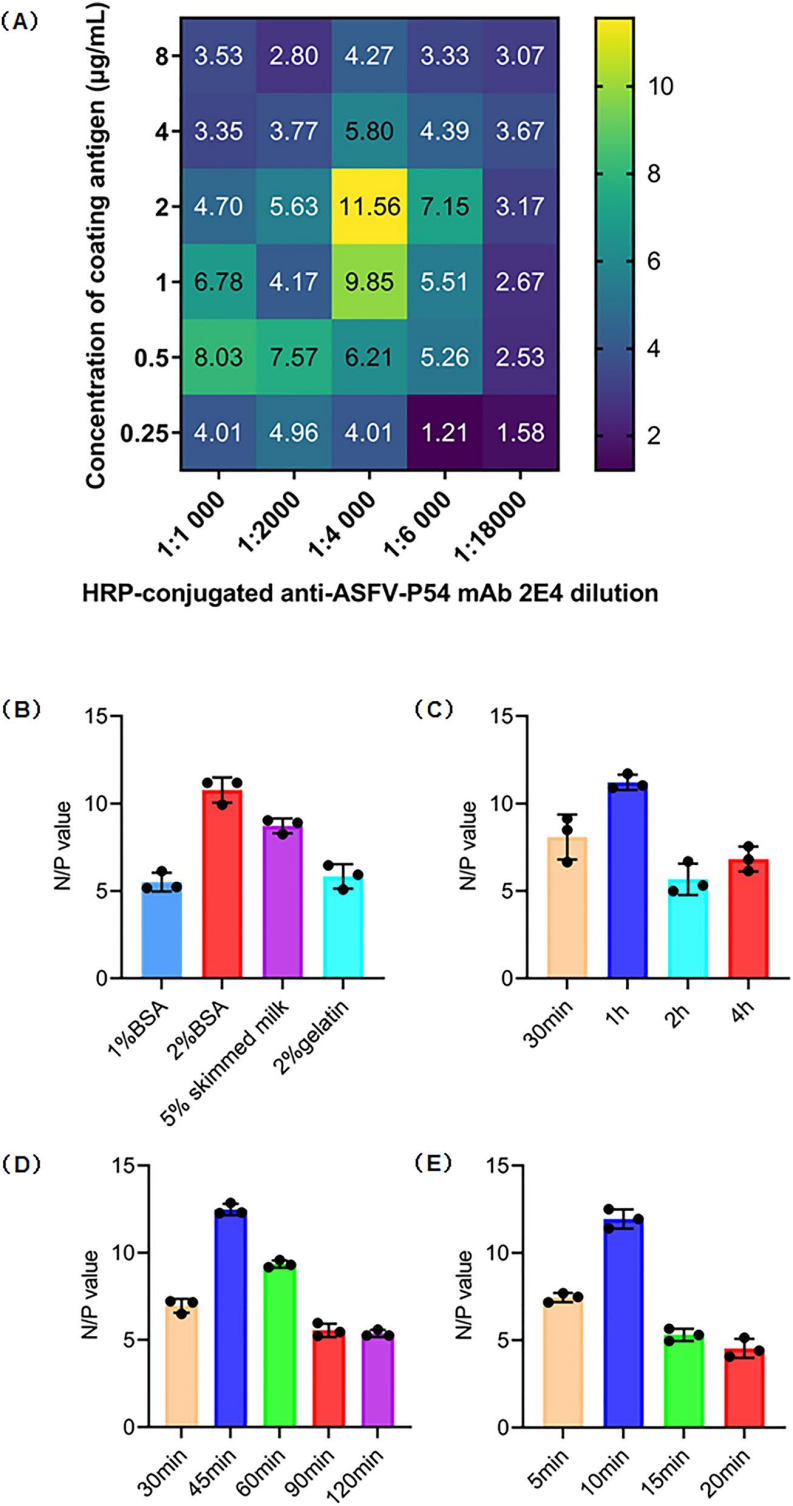
Optimization of the chimeric CLPs-based blocking ELISA. (**A**) Determination of the optimal working concentration of coating antigen and mAb 2E4 by checkerboard titrations. The optical density at 450 nm (OD_450nm_); ratios of negative to positive reference serum (N/P) are presented in a heatmap drawn by the GraphPad Prism software (version 9). The lighter the color, the greater the N/P OD_450nm_ ratio. (**B**) Comparison of the blocking effect of four blocking buffers (1% BSA, 2% BSA, 5% skimmed milk and 2% gelatin). (**C**) Determination of the optimal incubation time for serum samples. (**D**) Determination of the optimal incubation time for HRP-ASFV-P54-mAb 2E4. (**E**) Determination of the optimal chromogenic reaction time. All results were presented as the mean ± SD of triplicate experiments. The black circles represent the N/P values of three independent runs.

To develop a blocking ELISA for serological diagnosis of ASFV, the diluted chimeric CLPs were used as coat-antigens, and the HRP-anti-P54-mAb 2E4 were used as detection antibodies. Based on the checkerboard titration results (Fig. 5A), the maximum N/P value was obtained when the chimeric CLPs concentration was 2.0 μg/mL, and the dilution ratio of HRP-conjugated mAb 2E4 was 1:4000. Thus, the optimal antigen-coating concentration and detection antibody dilution of the developed blocking ELISA was determined. Additionally, other important conditions of the blocking ELISA, including blocking buffers, serum incubation time, HRP-mAb incubation time, and chromogenic reaction time, were also optimized. As illustrated in Fig. 5B, 2% BSA in PBST exhibited the best coating effect among the four blocking buffers tested; Fig. 5C showed that the optimal time of serum samples incubation was 1 h; Fig. 5D showed that the optimal reaction time of the HRP-ASFV-P54-mAb 2E4 was 45 min; and Fig. 5E indicated that the optimal chromogenic reaction time was 10 min.

### Determination of the optimal cut-off value, diagnostic specificity and sensitivity

**Figure 6:**
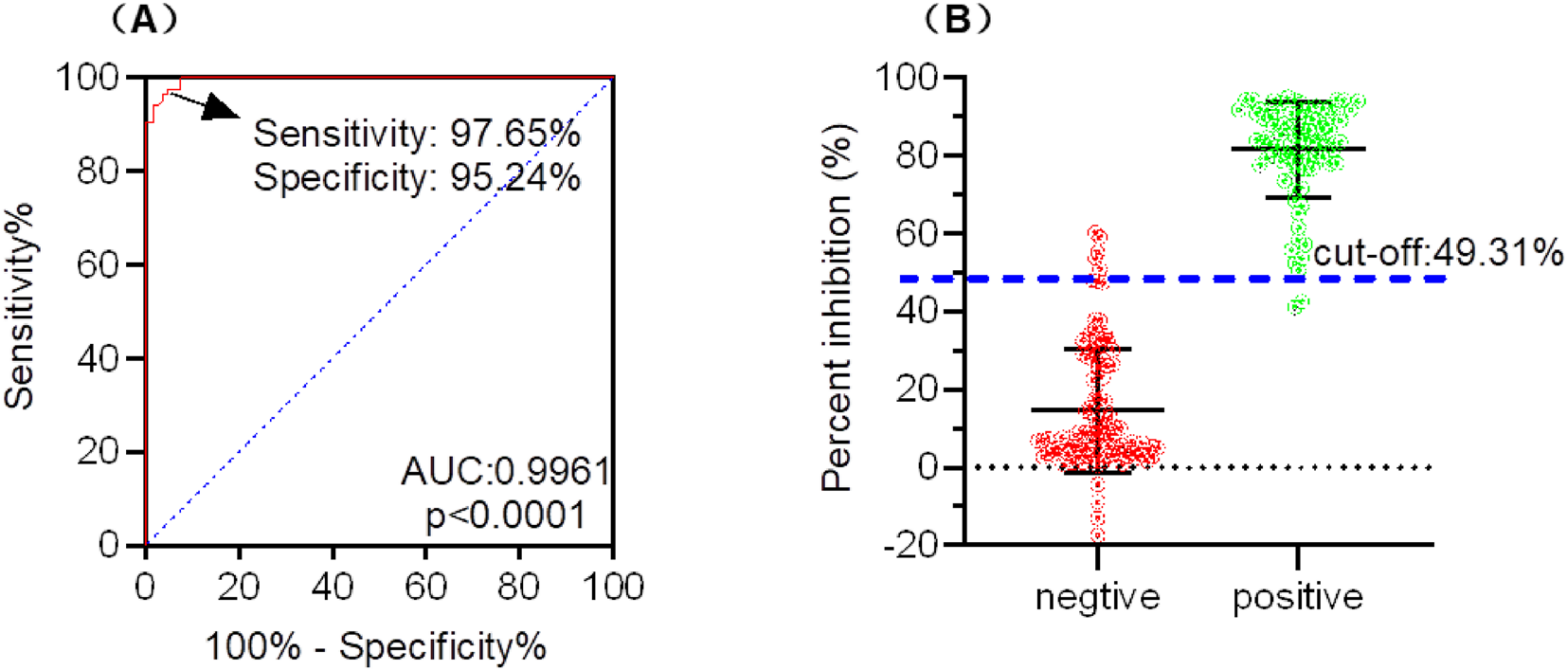
Cut-off value determination, diagnostic sensitivity and specificity analysis of the blocking ELISA based on chimeric CLPs. There were 105 ASFV negative serum samples and 85 ASFV positive serum samples detected by the developed blocking ELISA. (**A**) ROC analysis of blocking ELISA results while the area under the curve (AUC) of the test was 0.9961. (**B**) Interactive dot plot diagram showing the blocking value of serum samples while the cut-off value was set to 49.31%.

To determine the cutoff value of the developed blocking ELISA, the 190 swine serum samples were collected from the Guangdong Province of China between December 2018 and June 2019, including 85 inactivated ASFV-positive sera and 105 ASFV-negative sera. All the samples were tested using the developed blocking ELISA, and each sample’s PI value was calculated. A ROC curve statistical analysis was performed to determine the PI cut-off value and to evaluate the diagnostic sensitivity and specificity of the assay (Fig. 6A). An interactive dot plot diagram with the PI value of these samples was shown in Fig. 6B. According to the ROC analysis, the area under the curve (AUC) was 0.9961 (95% confidence interval: 0.9918 to 1.000). Besides, when the PI cutoff value was set to 49.31% for the developed blocking ELISA, the diagnostic sensitivity and specificity were 97.65% and 95.24%, and the corresponding Youden index (0.9765 + 0.9524 − 1 = 0.9288) was achieved to the maximum.

### Analytic specificity and sensitivity of the chimeric CLPs-based blocking ELISA

**Figure 7:**
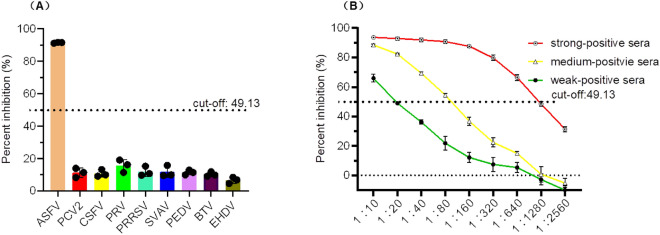
Analytic specificity and sensitivity of the developed the developed blocking ELISA. (**A**) Percent inhibition of the polyclonal anti-sera against various e viruses detected by the developed blocking ELISA. Only the PI value of ASFV positive sera was higher than the cut-off value. (**B**) Two-fold serially diluted ASFV strong-positive sera, medium-positive sera and weak-positive sera ranging from 1:10 to 1:2560 were detected via the developed blocking ELISA.

For assessing the analytic specificity, the developed blocking ELISA based on BTV CLPs chimerized with an antigenic epitope of ASFV P54 was used to detect several polyclonal anti-sera against other viruses, including PCV2, CSFV, PRV, PRRSV, SVAV, PEDV, BTV, and EHDV. As shown in Fig. 7A, the PI values of those sera were significantly lower than the cut-off value of the developed blocking ELISA, thereby indicating that these sera tested negative. These results suggested that the chimeric CLPs-based blocking ELISA was highly specific for the detection of ASFV, without exhibiting any cross-reactivity towards other virus-positive sera.

The analytic sensitivity of the chimeric CLPs-based blocking ELISA was evaluated using three Two-fold serially diluted ASFV positive (including strong-positive sera, medium-positive sera, and weak-positive sera) ranging from 1:10 to 1:2560. As shown in Fig. 7B, the maximum dilution of ASFV strong-positive sera, ASFV medium-positive sera, and ASFV weak-positive sera were 1:640, 1:80 and 1:10 respectively, which were detected to be positive. Thus, the blocking ELISA had a good analytical sensitivity.

### Repeatability and reproducibility of the chimeric CLPs-based blocking ELISA

To evaluate the repeatability and reproducibility of the ELISA, three ASFV positive sera (strong-positive sera, medium-positive sera, and weak-positive sera) and three ASFV negative sera were tested with this method on one plate in one run or on three plates in three independent runs. As shown in Table 1, the intra-assay CV of the PI ranged from 0.96% to 6.11%, and the inter-assay CV of the PI ranged from 1.05 to 9.14%, indicating a good repeatability and reproducibility of the chimeric CLPs-based blocking ELISA.

**Table 1:** Repeatability and reproducibility analysis of the developed blocking ELISA.

Samples	Repeatability (Intra-assay)			Reproducibility (Inter-assay)		
	Mean PI (%)	SD	CV	Mean PI (%)	SD	CV (%)
Strong-positive	91.23	0.0088	0.96	92.17	0.0096	1.05
Medium-positive	84.75	0.0123	1.45	81.70	0.0206	2.52
Weak-positive	65.21	0.0075	1.14	63.88	0.0124	1.94
Negative No.1	14.03	0.0086	6.11	16.17	0.013	8.06
Negative No.2	37.66	0.0168	4.46	38.21	0.0067	1.74
Negative No.3	5.50	0.0024	4.37	4.68	0.0043	9.14

PI Mean: the average of PI values from three repeated blocking ELISA detections; CV: coefficient of variation; SD: standard deviation.

### Agreements of the blocking ELISA with western blot and commercial ELISA kit results

To compare the conformance of the three methods, 493 inactivated pig sera were detected in parallel by the developed blocking ELISA, the western blot, and the commercial c-ELISA kit. As shown in Table 2, the compliance rates of blocking ELISA versus western blot and blocking ELISA versus commercial ELISA kit were 96.2% (475/493) and 97.76% (482/493). Statistical analysis showed that the Kappa value between the blocking ELISA and the western blot was 0.911, and the Kappa value between the blocking ELISA and the commercial ELISA kit was 0.946 (Table 2). Compared with the western blot, the sensitivity was 97.79%, the specificity was 95.79%, the positive predictive value was 89.86%, and the negative predictive value was 99.13%. Compared with the commercial ELISA kit, the sensitivity was 100%, the specificity was 96.11%, the positive predictive value was 92.56%, and the negative predictive value was 100%. Statistical analysis indicated that the established blocking ELISA had a high level of consistency with the western blot and the commercial kits.

**Table 2:** Results of field samples.

The blocking ELISA		Compared methods		Agreement (%)	Kappa value
		Western blot		96.35	0.911
		+	−		
+	148	133	15		
−	345	3	342		
		Commercial ELISA kit		97.76	0.946
		+	−		
+	148	137	11		
−	345	0	345		

## Discussion

ASF is a highly destructive disease that is causing severe economic and production losses to pig populations around the world. Due to the complex biologic characteristics of ASFV, there are currently no effective commercial vaccines or antivirus drugs against ASFV. As a result, Surveillance and diagnosis of pig populations have become critical to deal with ASF outbreaks. In the early stage of the outbreak of ASF, molecular biological methods such as PCR and real-time PCR were usually used to detect ASFV, as infected pigs often presented with acute or sub-acute infections with a mortality rate of up to 100%. However, as ASF continues to be prevalent in a region, ASFV maybe happens to mutate, and the number of pigs with recessive infection or surviving from ASFV infection would keep increasing^42,43^. In several areas of Africa and Europe, there were many pigs or wild boars that survived infection and presented no clinical signs of ASFV without the presence of ASFV attenuated variants^44–46^. In this situation, an accurate serological assay for detecting antibodies of the animals was needed to screen out pigs with recessive or asymptomatic infections. In addition, with the progress of technology, it is believed that an effective vaccine against ASF will be developed and widely used in the near future^47^. Once the pigs are immunized, the most direct way to reflect the immune effect is antibody detection. Therefore, a sensitive and reliable serological diagnostic assay of ASFV is required.

ASF serological detection assays mainly include indirect ELISA, blocking ELISA, competitive ELISA, and indirect immunofluorescence. The principle of blocking ELISA is that virus-specific antibodies in serum samples react with antigens to block the binding of an HRP-mAb to the antigens. The character of the monoclonal antibody used plays a crucial role in determining the specificity and other attributes of the blocking ELISA method. The choice of the appropriate monoclonal antibody is essential for developing a successful ELISA method that will provide high-level specificity and sensitivity. In this study, the HRP-conjugate mAb involved in the established blocking ELISA was anti-ASFV-P54-mAb 2E4. The antigenic epitope recognized by mAb 2E4 was mapped to be located at a highly conserved region between amino acids 66 and 74 (DIQFINPYQ) of ASFV P54 protein. The stability of binding between mAb 2E4 and the corresponding antigenic epitope polypeptide (DIQFINPYQ) was analyzed by a macromolecular interaction system (Bio-Rad, ProteOn XPR36). The results indicated that mAb 2E4 had a strong affinity with the polypeptide and could form a stable bond^33^. Therefore, mAb 2E4 is an ideal monoclonal antibody to develop a blocking ELISA for the serological detection of ASF.

For the construction of CLPs chimerized with an antigenic epitope of ASFV P54, we analyzed the structural protein vp3 and vp7 of BTV. One copy of CLP contains 780 copies of VP7, and trimers of VP7 are located at the outer surface of BTV CLPs like a spikey coat. Thus, compared with VP3, VP7 is more suitable as the target protein for displaying heterologous epitopes. In addition, researchers analyzed the crystal structure and epitopes of VP7 protein, revealed the role of VP7 in the self-assembly of BTV CLPs, and found several key amino acid residues of VP7, which affect the structure of CLPs^30,38,48,49^. Based on the previous studies, this study used an online analysis website to analyze the secondary structure of VP7 protein and selected four flexible structure areas of VP7 as the insertion sites of the epitope recognized by mAb 2E4. The results of our study showed that the flexible region of VP7 protein between aa259 and aa264 was a suitable position which inserted a short peptide (DIQFINPYQ) recognized by anti-ASFV-P54 mAb 2E4.

As a hollow particle similar to a virus, VLPs/CLPs do not contain the viral nucleic acid genome to be not infectious. Therefore, compared to whole or inactivated viruses, VLPs/CLPs are used as coating antigens can avoid virus transmission and reduce biosafety risks. At the same time, each VLPs/CLPs typically consists of hundreds of subunits. The BTV CLP consists of 120 copies of VP3 and 780 copies of VP7, and the 780 copies of VP7 protein like a brush coat on the outer layer of BTV CLPs. Therefore, compared to using the ASFV P54 protein obtained through prokaryotic or eukaryotic expression as the coating antigen, BTV CLPs could more and better display the antigenic epitope of ASFV P54, which is conducive to antibody binding, thus further improving the sensitivity and specificity of ELISA. In this study, we first used BTV CLPs chimerized with an antigenic epitope of ASFV P54 as the coating antigen and used the corresponding mAb as the detection antibody to develop a blocking ELISA for detecting ASFV antibodies. Through TEM observation, it was found that the structure and morphology of CLPs were destroyed when diluted with 0.05 M pH9.6 carbonate buffer as the coating solution, and the number of intact CLPs was significantly reduced. To ensure that the CLPs remained intact, this study used 0.2 M pH8.0 Tris–HCl buffer as the coating solution. A series of validation experiments showed that this method has good detection performance. By calculation, the optimal PI cut-off value of the blocking ELISA was determined to be 49.31%, which corresponded to a diagnostic sensitivity of 97.65% and a diagnostic specificity of 95.24%. The specificity tests performed that the chimeric CLPs-based blocking ELISA distinguished ASFV from other common swine viruses (CSFV, PCV2, PRRSV, SVAV, and PEDV) and the Orbivirus such as BTV and EHDV. The maximum dilution of the ASFV strong-positive sera, medium-positive sera, and weak-positive sera were 1:640 1:80, and 1:10, indicating that the chimeric CLPs-based blocking ELISA was high sensitivity. In the repeatability and reproducibility analysis, intra-assay and inter-assay CV both were < 10%, meaning that the chimeric CLPs-based blocking ELISA had adequate repeatability. This study did not compare the method with other ELISA methods using intact P54 protein or P54 partial peptides as coating antigens, so it was not possible to further determine the superiority of using CLPs to display P54 antigen epitopes. However, the blocking ELISA method established in this study showed high consistency with commercial kits and Western blot methods, indicating that using CLPs as an antigen epitope display platform to fully display foreign antigen epitopes in a form similar to virus structure can be used for ELISA or other serological detection methods.

## Conclusion

We successfully prepared chimeric CLPs with ASFV P54 epitope and established a blocking ELISA for detecting AFSV antibodies based on the chimeric CLPs and mAb 2E4 against ASFV P54. The blocking ELISA exhibited excellent repeatability in the detection of ASFV antibodies and did not cross-react with antisera against other pathogens. To our knowledge, this is the first report on the development of a blocking ELISA based on VLPs chimerized with an antigenic epitope of ASFV P54.

### Supplementary Information

Supplementary Information 1.

Supplementary Information 2.

Supplementary Information 3.

Supplementary Information 4.

Supplementary Information 5.

Supplementary Information 6.

Supplementary Information 7.

Supplementary Information 8.

## Data Availability

All data generated or analyzed during this study are included in the article and the additional file.
