# Supplementary figures and images for: A blocking ELISA based on virus-like nanoparticles chimerized with an antigenic epitope of ASFV P54 for detecting ASFV antibodies
Source: Sci Rep. 2023 Nov 15;13:19928. doi: 10.1038/s41598-023-47068-x (PMC10651890; doi:10.1038/s41598-023-47068-x)

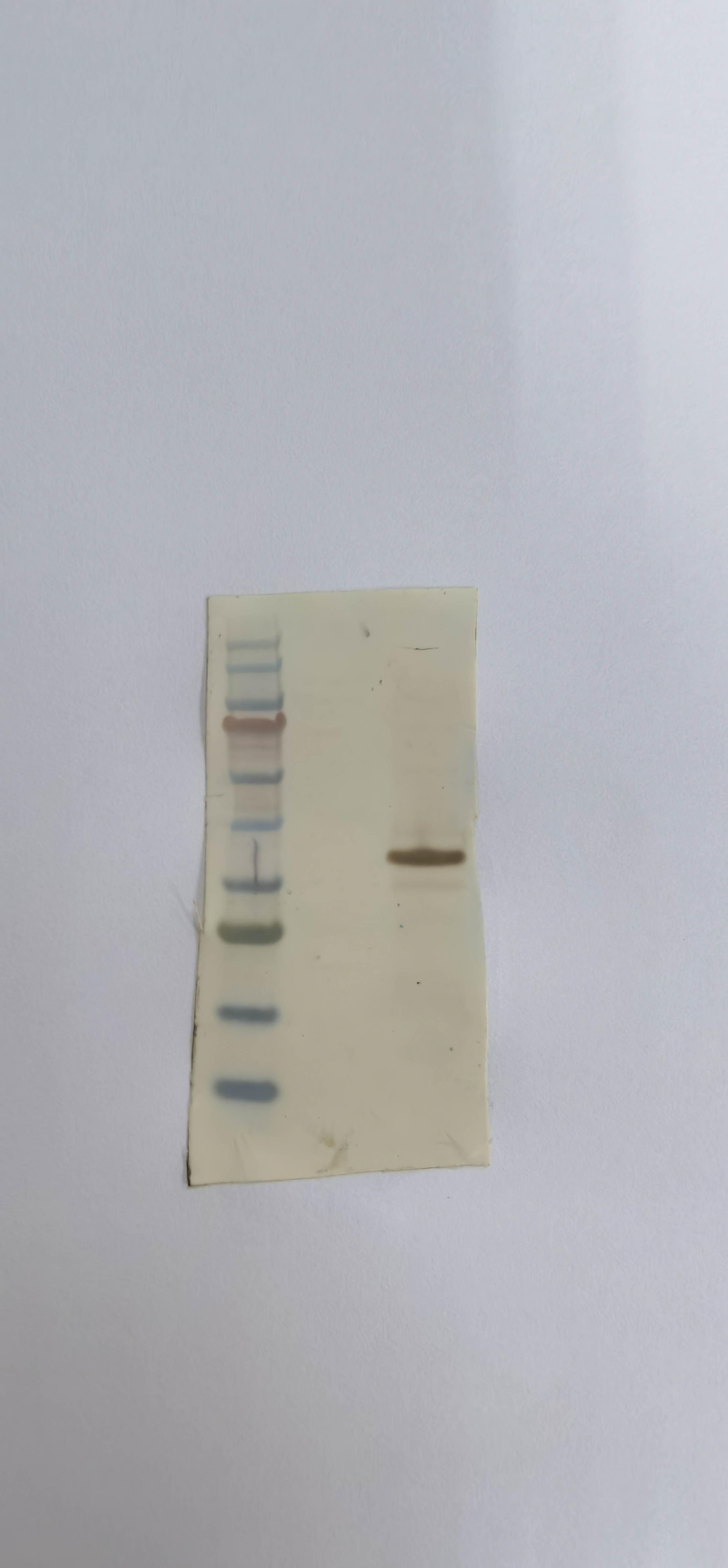

Supplement: Supplementary file 1 — Supplementary Information 1. [file 41598_2023_47068_MOESM1_ESM.jpg]

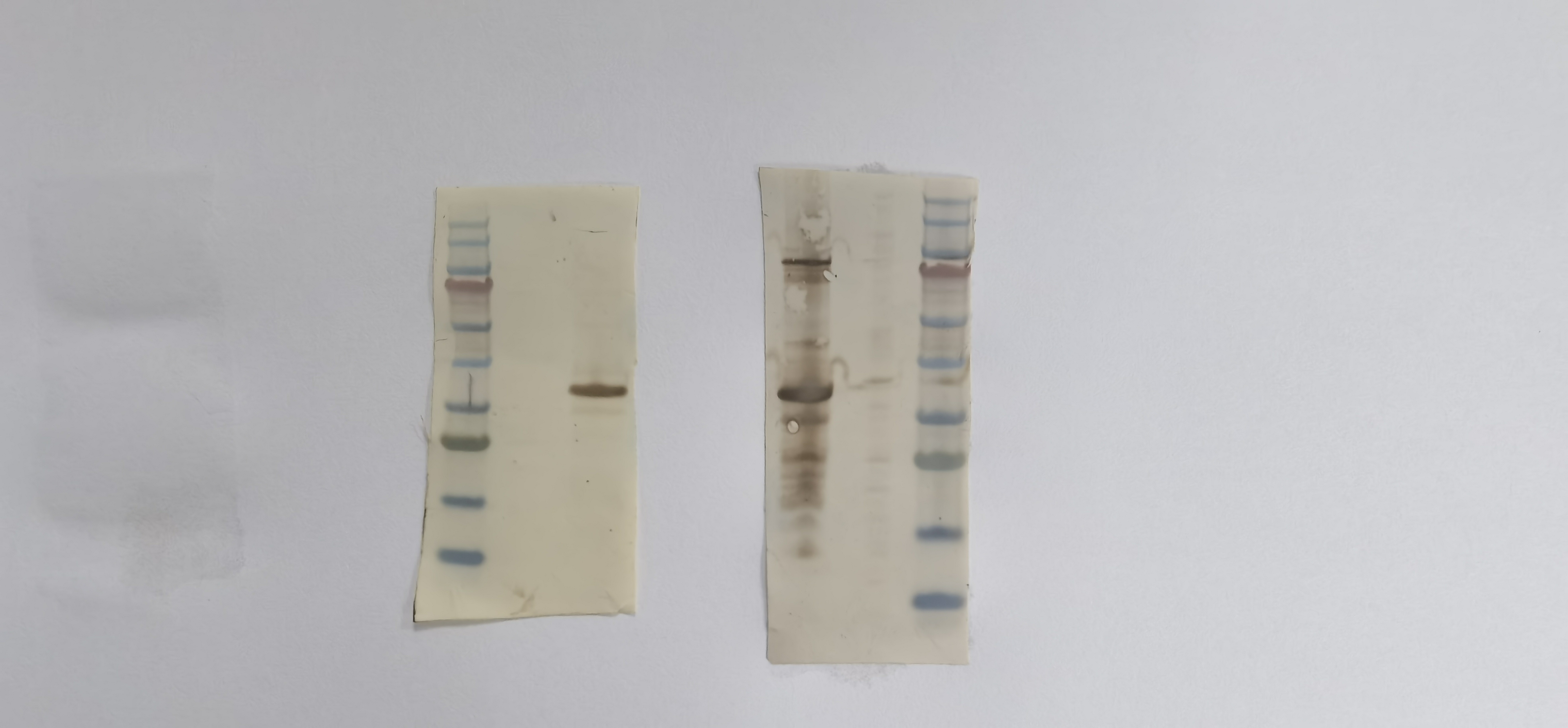

Supplement: Supplementary file 2 — Supplementary Information 2. [file 41598_2023_47068_MOESM2_ESM.jpg]

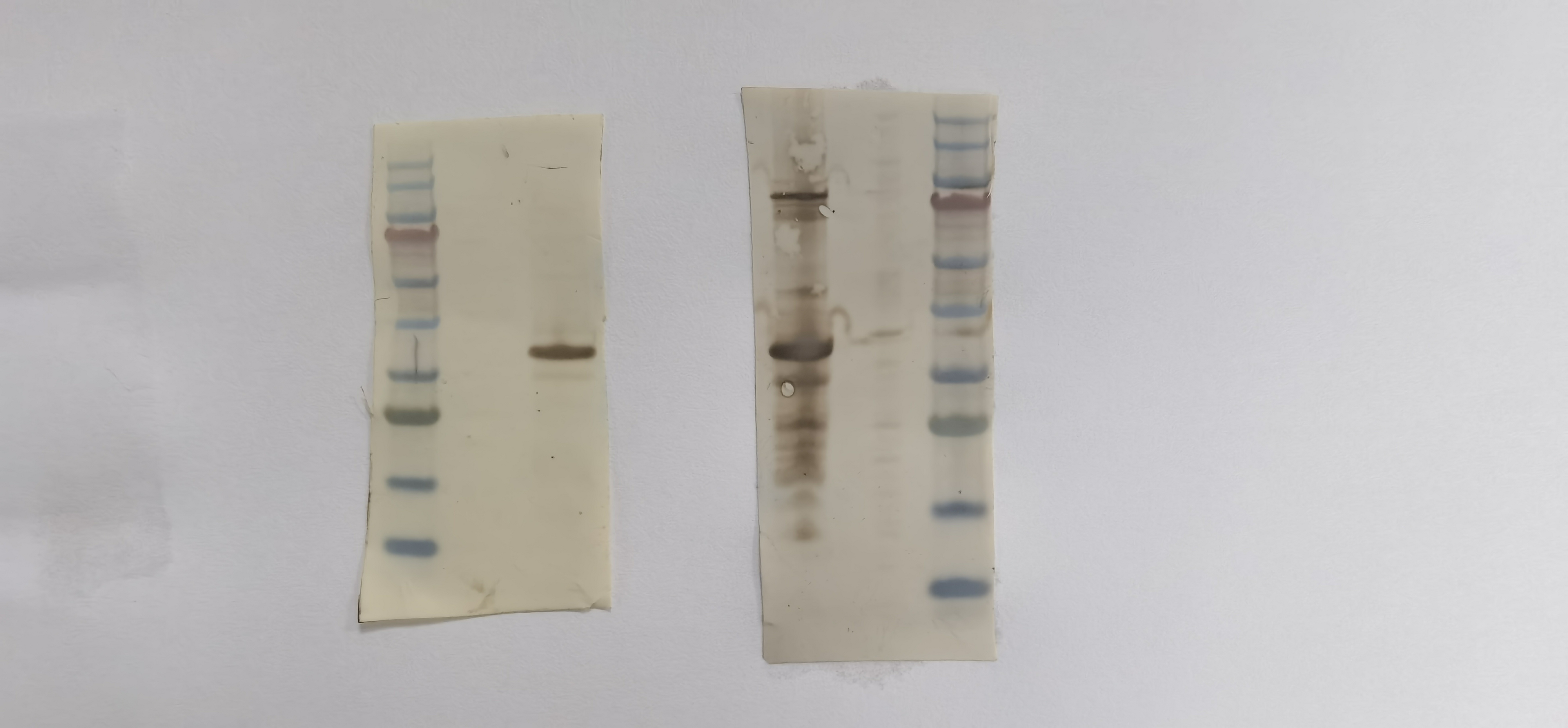

Supplement: Supplementary file 3 — Supplementary Information 3. [file 41598_2023_47068_MOESM3_ESM.jpg]

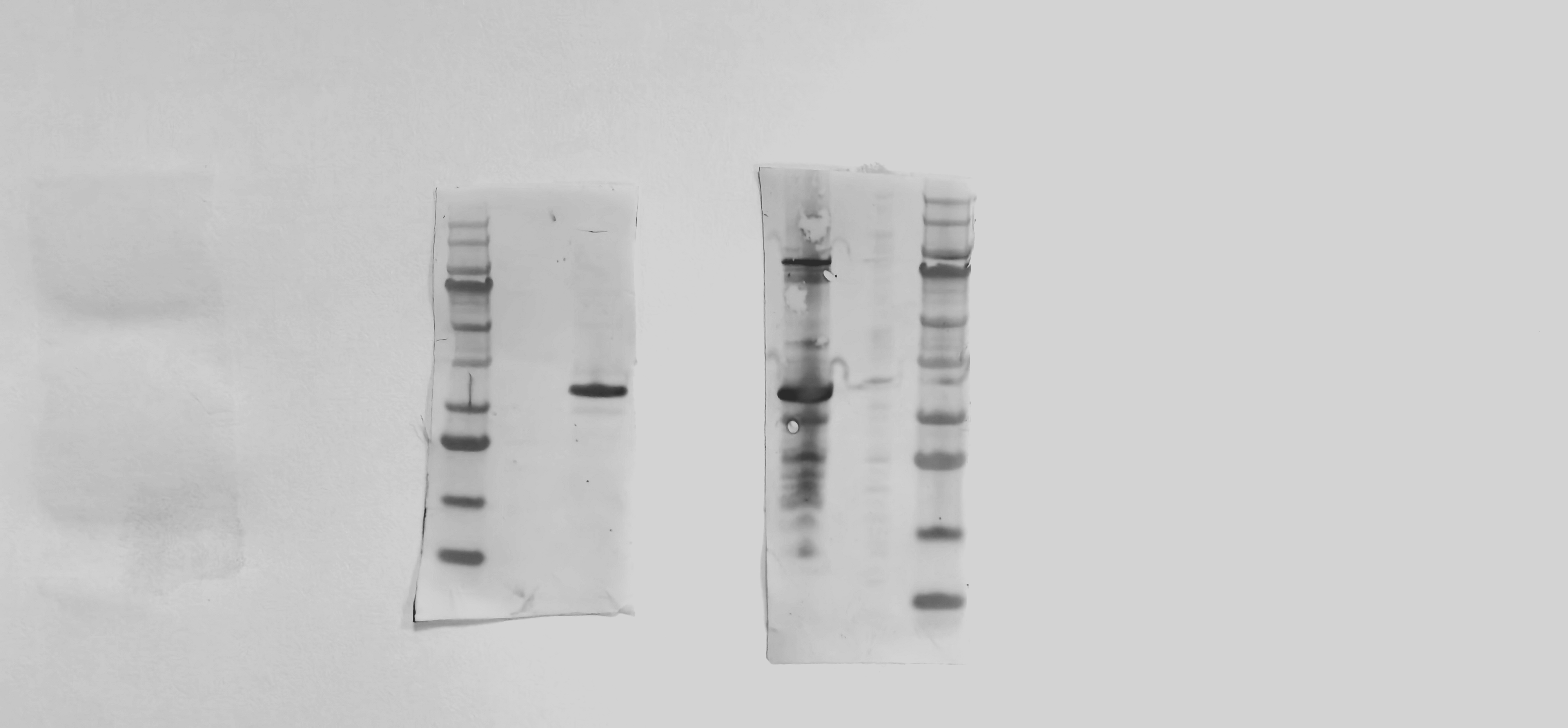

Supplement: Supplementary file 4 — Supplementary Information 4. [file 41598_2023_47068_MOESM4_ESM.jpg]

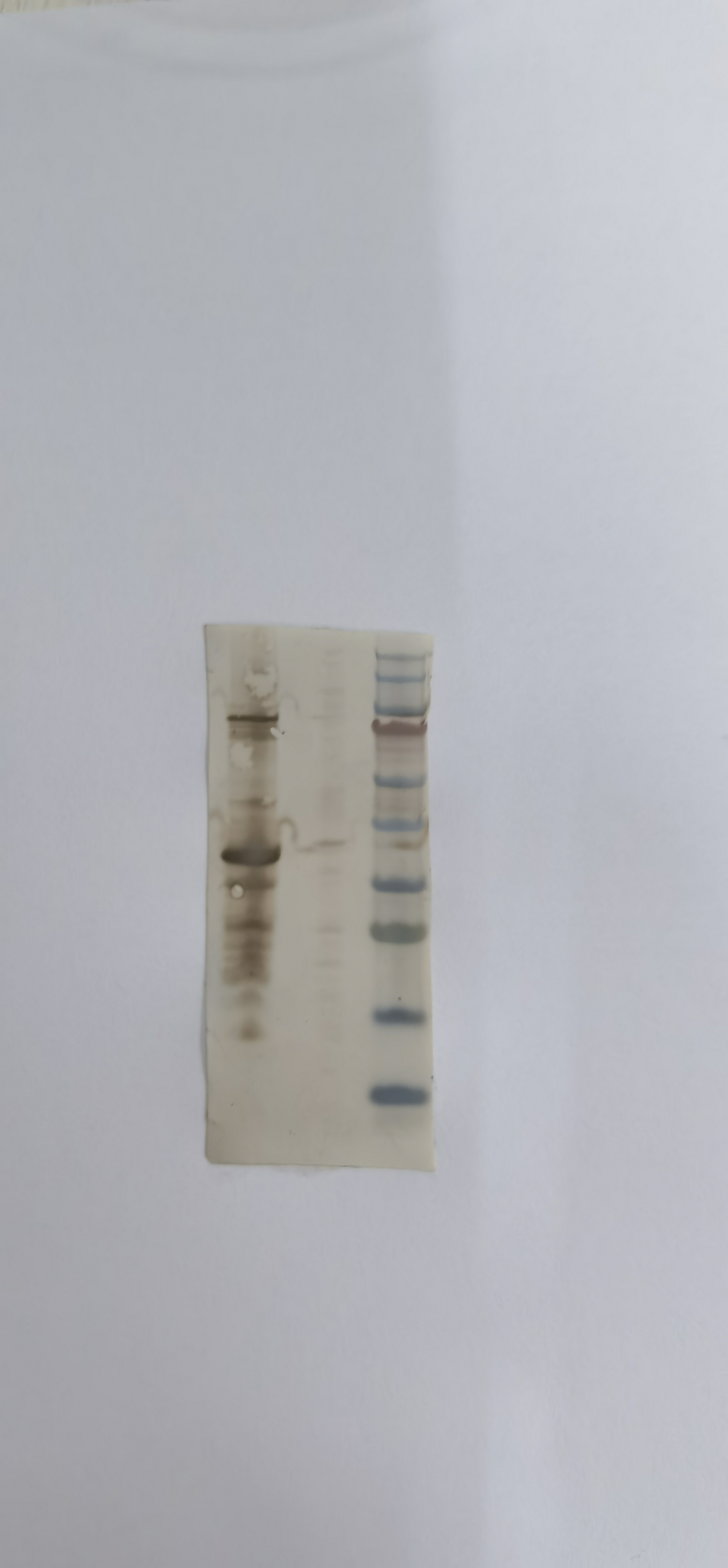

Supplement: Supplementary file 5 — Supplementary Information 5. [file 41598_2023_47068_MOESM5_ESM.jpg]
