# Supplementary material for: A blocking ELISA based on virus-like nanoparticles chimerized with an antigenic epitope of ASFV P54 for detecting ASFV antibodies
Source: Sci Rep. 2023 Nov 15;13:19928. doi: 10.1038/s41598-023-47068-x (PMC10651890; doi:10.1038/s41598-023-47068-x)
Supplement: Supplementary file 6 — Supplementary Information 6. [file 41598_2023_47068_MOESM6_ESM.docx]

Figure S1 The circle map of recombinant baculovirus transfer plasmid pFastBac-Dual-vp3-rvp7. In the recombinant baculovirus transfer plasmid pFastBac-Dual-vp3-rvp7, BTV VP3 was cloned into the *Nco* I-*Nhe* I restriction sites of the vector pFastBac^TM^Dual, and the rVP7 chimerized with an epitope of AFSV P54 was cloned into the *Not* I-*Sac* I restriction sites of the vector pFastBac^TM^Dual
