# Supplementary material for: A blocking ELISA based on virus-like nanoparticles chimerized with an antigenic epitope of ASFV P54 for detecting ASFV antibodies
Source: Sci Rep. 2023 Nov 15;13:19928. doi: 10.1038/s41598-023-47068-x (PMC10651890; doi:10.1038/s41598-023-47068-x)
Supplement: Supplementary file 7 — Supplementary Information 7. [file 41598_2023_47068_MOESM7_ESM.docx]

Supplement 2 The amino acid sequence of rVP7. A short peptide (DIQFINPYQ) recognized by anti-ASFV-P54 mAb 2E4 was inserted into the flexible region between aa259 and aa260 of the BTV VP7 protein

MDTIAARALTVMRACATLQEARIVLEANVMEILGIAINRYNGLTLRGVTMRPTSLAQRNEMFFMCLDMMLSAAGINVGPISPDYTQHMATIGVLATPEIPFTTEAANEIARVTGETSTWGPARQPYGFFLETEEVYQPGRWFMRAAQVVTPVVCGPDMIQVSLNAGARGDVQQIFQGRNDPMMIYLVWRRIENFSMPQGNSQRTLAGVTVSVGGVDMRAGRIIAWDGQAVLQIHNPTQQNAMVQIQVVFYISMDKTLNQDIQFINPYQYPALTAEIFNVYSFRDHTWHGLRTAILNRTTLPNMLPPIFPPNDRDSILTILLLSTLADVYSVLRPEFAIHGVNPMPGPLTRAIARAAYA-
