# Supplementary material for: A blocking ELISA based on virus-like nanoparticles chimerized with an antigenic epitope of ASFV P54 for detecting ASFV antibodies
Source: Sci Rep. 2023 Nov 15;13:19928. doi: 10.1038/s41598-023-47068-x (PMC10651890; doi:10.1038/s41598-023-47068-x)
Supplement: Supplementary file 8 — Supplementary Information 8. [file 41598_2023_47068_MOESM8_ESM.docx]

Supplement 3 The DNA sequence of rVP7. A nucleic acid sequence of an epitope (DIQFINPYQ) recognized by anti-ASFV-P54 mAb 2E4 was located between 778 bp~ 804 bp of the DNA sequence of the rVP7 chimerized with an epitope of ASFV-P54

ATGGACACTATCGCTGCAAGAGCACTCACTGTGATGCGGGCATGTGCCACGTTACAAGAAGCAAGAATTGTATTAGAAGCGAATGTAATGGAAATTTTGGGAATAGCTATCAATAGGTATAATGGATTGACTTTGCGTGGAGTCACGATGAGGCCCACCTCTCTAGCTCAAAGGAATGAGATGTTTTTCATGTGTTTAGACATGATGTTGTCAGCTGCTGGAATTAATGTTGGACCAATATCGCCAGATTATACCCAACATATGGCTACCATCGGTGTTTTAGCGACTCCAGAGATACCATTTACAACTGAGGCGGCGAATGAGATTGCGCGTGTAACTGGAGAAACTTCAACTTGGGGACCGGCTCGTCAGCCGTATGGTTTCTTCCTCGAGACGGAGGAGGTGTATCAACCGGGAAGATGGTTCATGAGAGCAGCTCAAGTGGTCACGCCTGTGGTTTGCGGCCCGGATATGATCCAGGTTTCATTGAACGCAGGAGCAAGAGGTGATGTACAGCAGATTTTTCAAGGTCGAAACGACCCGATGATGATTTATCTGGTTTGGAGAAGGATTGAAAATTTTTCTATGCCTCAAGGTAACTCACAGCGGACGTTAGCAGGAGTGACAGTAAGTGTAGGGGGTGTGGATATGAGGGCAGGACGGATAATTGCATGGGATGGACAGGCGGTTCTGCAGATACATAATCCTACTCAACAGAATGCAATGGTACAGATTCAAGTTGTGTTTTATATTTCAATGGATAAGACGCTAAATCAAGATATACAGTTTATAAATCCTTATCAATATCCAGCTCTGACAGCAGAGATTTTTAATGTATATAGTTTCAGAGACCACACATGGCATGGATTAAGAACGGCAATACTGAATAGAACAACCTTACCAAACATGCTTCCACCGATTTTTCCACCAAATGATAGAGATAGCATCCTAACAATCTTACTACTGTCGACGCTCGCGGATGTTTATTCGGTGTTGAGGCCTGAGTTTGCAATACACGGTGTAAATCCAATGCCTGGTCCGCTCACACGTGCAATTGCACGCGCCGCTTACGCATAG
